# Identifying Host Molecular Features Strongly Linked With Responses to Huanglongbing Disease in Citrus Leaves

**DOI:** 10.3389/fpls.2018.00277

**Published:** 2018-02-28

**Authors:** Bipin Balan, Ana M. Ibáñez, Abhaya M. Dandekar, Tiziano Caruso, Federico Martinelli

**Affiliations:** ^1^Dipartimento di Scienze Agrarie, Alimentari e Forestali, Università degli Studi di Palermo, Palermo, Italy; ^2^Department of Plant Sciences, University of California, Davis, Davis, CA, United States

**Keywords:** Huanglongbing, HLB, citrus, protein–protein interaction network, transcriptomics, RNA-Seq

## Abstract

A bioinformatic analysis of previously published RNA-Seq studies on Huanglongbing (HLB) response and tolerance in leaf tissues was performed. The aim was to identify genes commonly modulated between studies and genes, pathways and gene set categories strongly associated with this devastating Citrus disease. Bioinformatic analysis of expression data of four datasets present in NCBI provided 46–68 million reads with an alignment percentage of 72.95–86.76%. Only 16 HLB-regulated genes were commonly identified between the three leaf datasets. Among them were key genes encoding proteins involved in cell wall modification such as CESA8, pectinesterase, expansin8, expansin beta 3.1, and a pectate lyase. Fourteen HLB-regulated genes were in common between all four datasets. Gene set enrichment analysis showed some different gene categories affected by HLB disease. Although sucrose and starch metabolism was highly linked with disease symptoms, different genes were significantly regulated depending on leaf growth and infection stages and experimental conditions. Histone-related transcription factors were highly affected by HLB in the analyzed RNA-Seq datasets. HLB tolerance was linked with induction of proteins involved in detoxification. Protein–protein interaction (PPI) network analysis confirmed a possible role for heat shock proteins in curbing disease progression.

## Introduction

The pathogenetic mechanisms of Huanglongbing (HLB) disease remain unclear. The disease is caused by a phloem-limited bacterium, Candidatus liberibacter asiaticus (CaLas), transmitted by psyllids. The pathogen has three subspecies: americanus, africanus, and asiaticus. The first two subspecies infect Citrus in South America and Africa, respectively, while the asiaticus subspecies is widespread in North America and Asia. The pathogen is closely related to Rhyzobiaceae and has biotrophic behavior. The caused disease is the most threatening in Citrus worldwide and leads to tree death in few years, reduced tree growth, yellowing of leaves and malformed, unmarketable fruits characterized by small seeds, high acidity, small size, and altered ripening dynamics.

Although genomic sequences have been determined (Duan et al., 2009; Tyler et al., 2009) and putative toxins have been isolated, the toxic molecules are not the only cause of disease symptoms. Previous -omic approaches have provided insight into the molecular mechanisms provoking symptoms. The first studies conducted through microarrays highlighted upregulation of genes involved in carbohydrate biosynthesis and metabolism, particularly those involved in starch pathways, such as AGPase and starch synthase (Albrecht and Bowman, 2008; Kim et al., 2009). Repression of photosynthesis and other primary metabolic pathways was also observed. RNA-Seq studies on different sink and source organs showed that sink-source tissue relationships were severely modified by the disease (Martinelli et al., 2012, 2013). Upregulation of glucose-phosphate-transporter2 was considered a key factor driving starch accumulation in infected leaves, a common symptom of the disease. CaLas-infected fruits remained green and photosynthesizing, while the mature leaves had decreased photosynthesis and yellowing due to starch accumulation, causing inversion of the usual relationship between developing fruit and mature leaves (Martinelli et al., 2015). Another important factor causing symptoms was reduced expression of genes encoding heat shock proteins (i.e., HSP82). The products of these genes protect protein folding and function during stress conditions. These genes help maintain normal function of key proteins, especially in phloem and leaves, maintaining correct function of proteins involved in primary metabolism. A third devastating effect of the pathogen is modified hormonal cross-talk. The pathogen induced upregulation of key genes involved in jasmonic acid-mediated responses in leaves (lox1, lox2, and lox3), probably in response to insect attacks. In addition, the salicylic acid-mediated response was more highly induced in fruits than in leaves, although fruits are not commonly the place of infection. Finally, induction of abscissic acid and auxin genes (HVA22C, SAUR-like, and UGT71B6) should counteract the action of salicylic acid responses, helping the pathogen grow and develop. Proteomic approaches performed to study pathogenetic mechanisms of the disease confirmed these findings, highlighting downregulation of photosynthesis-related proteins and modification of transport, carbohydrate metabolism, hormone biosynthesis, metabolism and xenobiotic responses. At the proteomic level, proteins involved in detoxification of oxidative stresses (glutathione S-transferases and nitrilases), in cell wall modification and pathogenesis-related processes were most effective in promoting citrus tolerance (Martinelli et al., 2016).

Studies of the transcriptome, proteome, and metabolome led to design of new translational genomic tools to speed diagnosis and develop short- and long-term therapeutic and genetic resistance strategies (Dandekar et al., 2010; Ibáñez et al., 2014). Newly developed sensor devices that capture early-induced molecules produced by infected leaves and fruits could distinguish recently infected, still-asymptomatic trees from severely symptomatic ones (Aksenov et al., 2014). This approach might be extended and applied to other specialty crops (Martinelli et al., 2016). Diagnostic methods focusing on host responses are highly desirable as a complement to traditional approaches targeting the pathogen, as the latter can seldom detect the pathogen before visual symptoms occur.

The -omic approaches do have weaknesses: low reliability and scarce or (often) absent experimental repetition. They are performed in different environments and seasons on trees grown under different agronomic conditions. There is an urgent need to perform duplicate field studies due to the many environmental variables that affect gene expression. The timing of disease progression from infection to tree death has also led to contradictory conclusions. Some of the issues can be addressed using meta-analysis to compare differentially regulated genes and affected pathways among different studies using the same bioinformatic methods (Rawat et al., 2015).

The aim of this study was to perform a bioinformatic analysis of previously published RNA-Seq studies on leaves of CaLas-infected citrus using the same pipeline. We analyzed the raw datasets using the most-updated bioinformatic pipeline and an integrated functional data mining approach to identify common molecular patterns that were consistently linked with pathogen infection.

## Materials and Methods

### Search Strategy to Identify Published Studies for Bioinformatic Analysis

**FIGURE 1:**
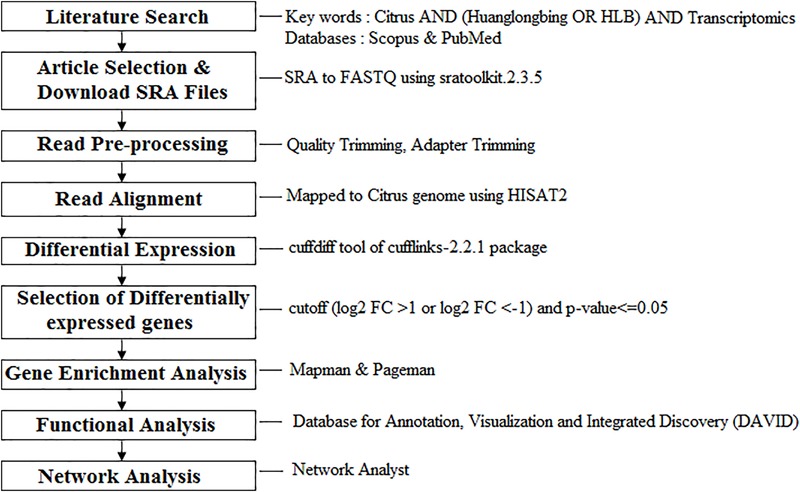
Meta-analysis workflow of the four RNA-Seq data dealing with Huanglongbing (HLB) response and tolerance in leaf tissues. Functional data mining tools were provided.

The published RNA-Seq studies in Citrus related to HLB response and tolerance in leaf tissues were searched using Scopus and PubMed. We found three studies published on or before May 2017 (Martinelli et al., 2013; Fu et al., 2016; Wang et al., 2016). We divided the first RNA-Seq study into two datasets for young and mature leaves (Martinelli et al., 2013). The second study was performed under controlled conditions using artificial infection of young leaves (Fu et al., 2016). The third work (Wang et al., 2016) compared Citrus tolerance mechanisms with the list of HLB-regulated genes in common between the previous two studies. We first analyzed the three transcriptomic datasets related to HLB-response. We next identified genes related to tolerance that were present in the three datasets dealing with HLB response. We downloaded raw data from the four datasets and performed our bioinformatics pipeline as described (**Figure 1**).

### Bioinformatic Analysis of Raw Data

The Citrus sinensis v1.1 and annotation file were downloaded from Phytozome^1^. The raw files (SRA format) of the three articles were downloaded from NCBI SRA and then converted to FASTQ format using SRAToolkit version 2.3.5. The raw reads were filtered to obtain high-quality clean reads by trimming low-quality bases followed by adaptor sequence removal using cutadapt version 1.8.1. The pre-processed reads were mapped to the Citrus sinensis genome v1.1 with HISAT2 version 2.0.5 (Kim et al., 2015) using default parameters. The identification of differentially expressed genes was performed using Cuffdiff algorithm in Cufflinks version 2.2.1 pipeline with default parameters (default false discovery rate, FDR is 0.05). The annotated cuffdiff result files of all comparisons including the *q*-value (the FDR-adjusted *p*-value of the test statistic) and significant (Can be either “yes” or “no”, depending on whether *p* is greater than the FDR after Benjamini-Hochberg correction for multiple-testing) of all comparisons were provided in Supplementary Table S3.

### Differentially Expressed Gene Selection and Splice Analysis

The up- and downregulated genes with fold change cutoff (log2 FC > 1 or log2 FC < -1) and *p*-value <0.05 were identified from the selected articles. Citrus sinensis gene ids were mapped to corresponding TAIR IDs using the mapping file downloaded from the Phytozome database^2^. The common and unique differentially HLB-regulated genes among different studies were identified. A custom made in-house Perl script was employed for gene selection and mapping.

The alternative splicing events of each samples were predicted by the ASTALAVISTA program (Foissac and Sammeth, 2007) on the web server^3^ using the GTF files generated by Cufflinks. Differential splicing analysis was done using MISO version 0.5.3^4^ (Katz et al., 2010) and rMATS version 4.0.1^5^ (Shen et al., 2012) using the default options. The Sashimi plots were generated in order to get the quantitative visualization of the aligned RNA-Seq reads which enables quantitative comparison of exon usage across the control and treated samples.

### Gene Enrichment and Functional Analysis

MapMan (Thimm et al., 2004) was used with the Citrus sinensis mapping file^6^ to map the gene ids and visualize the metabolic overview, large enzyme families, hormone-related genes, transcription factors and biotic-stress related genes in all four transcriptomic datasets. PageMan (Usadel et al., 2006) analysis was used for gene set enrichment analysis and to visualize differences among metabolic pathways using Wilcoxon tests, no correction, and an over-representation analysis (ORA) cutoff value of 1.

Pathway enrichment analysis using gene ontologies was conducted using Database for Annotation, Visualization and Integrated Discovery (DAVID; Web server^7^) (Huang et al., 2009). The complete DAVID pathway search results were provided. The gene ontology information related to Biological process was downloaded from the DAVID results (FDR cutoff = 0.05).

### Protein–Protein Interaction Network

Individual data annotation and analysis were performed using NetworkAnalyst (Xia et al., 2014), a web-based tool to visualize protein–protein network analysis. The homologous TAIR IDs were uploaded and mapped against the STRING interactome database using default parameters provided in NetworkAnalyst. We selected ‘Minimum Network’ to simplify the network and highlight key connections. First, we performed network analysis between the two transcriptomic datasets using the same tissue (young leaves). Next, we compared the common HLB-regulated genes (Martinelli et al., 2013; Fu et al., 2016) with those linked to tolerance (Wang et al., 2016).

## Results

### Workflow, Bioinformatics Analysis, and Venn Diagrams

Twelve RNA-Seq raw datasets from three published articles were analyzed using a bioinformatic and functional data mining pipeline (**Figure 1** and **Table 1**). The first study had two pairwise comparisons between apparently healthy and symptomatic young and mature leaf samples (Martinelli et al., 2013). Trees grew in the same orchard and were both infected by CaLas. Raw data submitted to NCBI were re-analyzed. Over 46–68 million reads were obtained for the four samples with an alignment percentage of 72.95–82.29%. The second study used a single pairwise comparison between healthy and infected leaf samples (Fu et al., 2016). The number of reads from the control sample was much less than that from the infected one, although alignment percentages were similar. This may reduce the depth of the transcriptomic analysis. The third study compared susceptible and tolerant Citrus genotypes to identify which genes were related to molecular mechanisms of tolerance in leaves (Wang et al., 2016). Three biological replicates were used and 72–118 million reads were obtained, with 73.42–82.44% alignment.

**Table 1:** Analyzed articles, SRA Ids, tissue, Read count, and alignment information.

Article	SRR ID	Original sample name	Sample tissue	Given sample name	Total reads	Alignment %
Martinelli et al., 2013	SRR867442, SRR867443	ML+AH	Mature leaves	Control-A	682,63,920	81.69
	SRR867449	ML+SY		Infected-A	625,03,214	72.95
	SRR867426	YL+AH	Young leaves	Control-B	641,86,912	82.29
	SRR867431	YL+SY		Infected-B	462,96,354	81.20
Fu et al., 2016	SRR3032893	Healthy(H)	Young leaves	Control-C	85,23,448	86.76
	SRR3032892	CaLas-B232		Infected-C	945,69,124	86.87
Wang et al., 2016	SRR2224205	R19T23	Young leaves	Susceptible	1061,89,916	78.84
	SRR2224296	R19T24			1183,00,964	75.65
	SRR2224411	R20T24			1018,35,958	75.13
	SRR2224421	R20T17		Tolerant	1128,32,326	73.42
	SRR2224429	R19T17			724,91,540	82.44
	SRR2224406	R20T18			1024,57,842	77.78

We identified 939 significantly CaLas-regulated genes in mature leaves from the first study: 516 upregulated and 423 downregulated from apparently healthy samples (Supplementary Table S1). In young leaves, 924 genes were significantly affected: 430 upregulated and 494 downregulated. In the third dataset, there were fewer repressed genes than CaLas-upregulated ones. The molecular study addressing the understanding of tolerance highlighted 3,469 significantly regulated genes: 1,712 upregulated and 1,757 downregulated in tolerant genotypes compared to susceptible ones.

**FIGURE 2:**
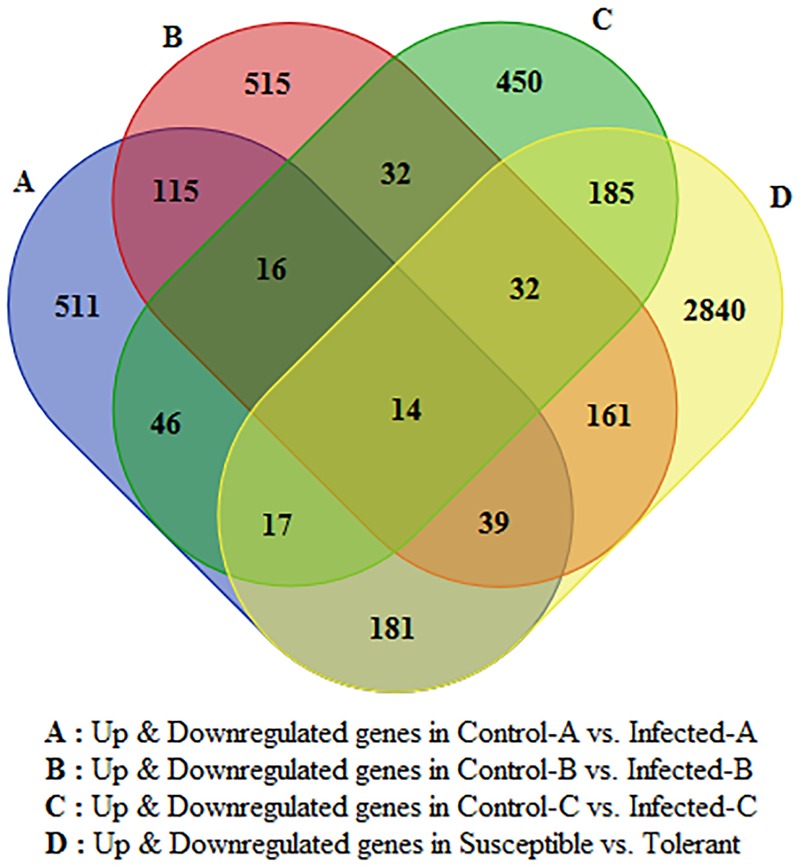
Venn diagram indicating number of HLB-regulated genes commonly modulated between the four datasets and specifically induced by HLB in each of the four RNA-Seq datasets.

Only 16 genes were commonly modulated between the three transcriptomic leaf datasets and 14 were commonly modulated in all four transcriptomic works (**Figure 2**). Between young and mature leaves infected in the field, 115 genes were commonly modulated by HLB. Five hundred fifteen were significantly regulated in young leaves and 511 in mature leaves. Six hundred twenty-nine genes linked with HLB tolerance were also modulated in the three leaf datasets.

### Gene Set- and Pathway-Enrichment Analysis

While in naturally HLB-infected mature leaves, no biological processes related to defense mechanisms were induced (Sample Name A), in young leaves, some key gene-ontologies were over-repressed in infected young leaves: defense response to bacterium, lipid oxidation, oxylipin biosynthesis, and response to salicylic acid (Sample Name B) (Supplementary Table S2). In artificially infected young leaves (Sample Name C), other gene set categories were affected such as jasmonic acid biosynthesis, plant-type hypersensitive response, and response to wounding. The expression patterns of all genes significantly regulated by CaLas identified in the three articles are shown (Supplementary Tables S3, S4). The complete gene set enrichment analysis using DAVID is shown.

### Molecular Responses to Huanglongbing Disease

#### Metabolism Overview

**FIGURE 3:**
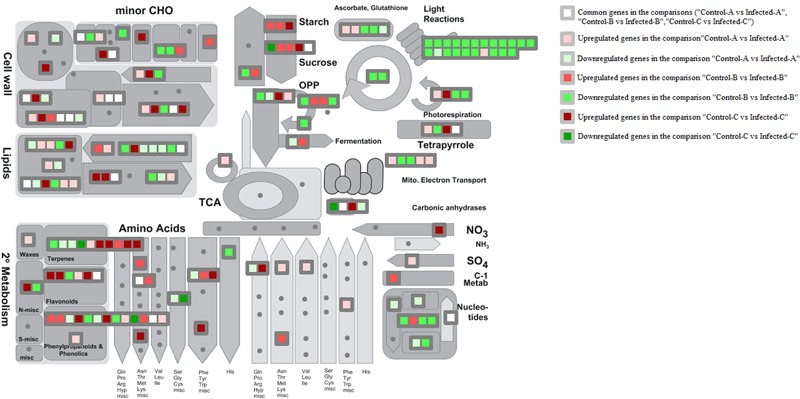
MapMan overview of three transcriptomic datasets related to HLB response. Up- and downregulated genes in the three pairwise comparisons were shown. Common HLB-regulated genes between at least 2 of 3 pairwise comparisons were also shown.

MapMan metabolism overview highlighted that HLB disease highly repressed photosynthesis in mature leaves and somewhat in young leaves (**Figure 3**). Upregulation of starch and sucrose metabolism was shown by the induction of key genes encoding beta-amylase6, glucan phosphorylase in mature leaves, and phosphoglucan water dikinase in young leaves at 32 weeks after infection (w.a.i.). A contrasting expression pattern was observed for different genes from the same pathway. Some genes were upregulated and others were repressed in primary metabolic pathways such as glycolysis, oxidative phosphate phosphorylation, fermentation, photorespiration, and tetrapyrrole. Trehalose-6-phosphate phosphatase and myo-inositol oxygenase were upregulated in young leaves after artificial infection while aldo/keto reductase and 1,3–beta glucan synthase were induced in mature leaves. Cell wall-related genes were highly affected in all three gene expression datasets. UDP-D-glucuronate-4-epimerase6, pectate lyase, RD22 nutrient reservoir, and pectin methylesterase were enhanced in young leaves at 32 w.a.i. Genes upregulated in young CaLas-infected leaves included UDP-glucose/UDP-galactose, 4-epimerase5, cellulose synthase, and the glycoside hydrolase 28 family. Lipid-related genes were particularly affected in immature leaves: choline kinase, glycerol-3-phosphate acyl transferase, and SUR4 membrane protein. Amino acid metabolism genes were mostly upregulated in young leaves at 32 w.a.i.: shikimate dehydrogenase, chorismate mutase, and homoserine dehydrogenase. Genes involved in amino acid biosynthesis were more induced in young leaves (L-asparaginase, alanine-glyoxylate aminotransferase, and enoyl-CoA hydratase).

Terpene-related genes encoding myrcene synthase, beta-amyrin synthase, and terpene synthase 21 were enhanced in young leaves 32 w.a.i., while homogentisate phytyltransferase 1 was repressed. Genes encoding chalcone and stilbene synthase and isoflavone reductase were upregulated in young leaves, while two methyltransferase family 2 proteins were induced in mature leaves.

Key genes involved in sucrose and starch metabolism were induced by CaLas in multiple studies. However, the same gene isoforms were not always affected. In mature leaves of naturally infected field trees, invertase1 and invertase2 were upregulated while beta-fructosidase4 was commonly modulated by different studies. Starch branching enzyme2, glucan phosphorylase, and beta-amylase6 were induced in mature leaves (Martinelli et al., 2013), while a phosphoglucan water dikinase was enhanced in young leaves after artificial infection (Fu et al., 2016).

#### Hormone Overview

**FIGURE 4:**
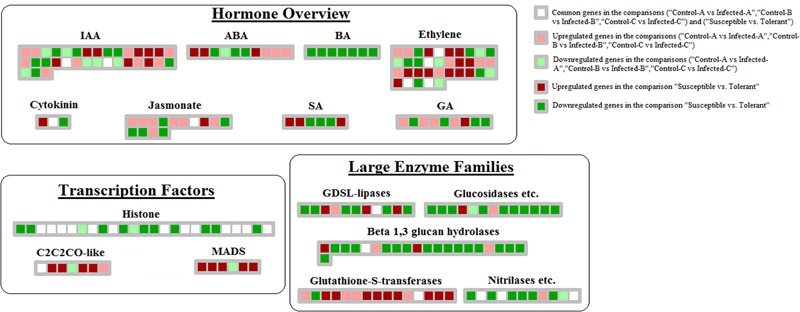
Genes linked with HLB tolerance and encoding transcription factors, enzymes, proteins involved in hormone-related pathways. Those genes commonly modulated between this dataset and the 3 related to HLB-response were shown.

Comparison between datasets showed that hormone crosstalk was severely modified by CaLas infection. Jasmonic acid-mediated response was highly induced in young leaves at 32 w.a.i.: lox1, lox2, and lox3 genes were upregulated (**Figure 4**). S-adenosylmethyltransferase was affected by HLB in multiple datasets. However, the induction of genes involved in auxin and abscissic acid synthesis might counteract the beneficial effects of this gene. Abscissic acid-response upregulated genes included GRAM-domain containing protein and benzodiazepine receptor-related, while auxin-related genes induced in young leaves included IAA-alanine resistant3, IAA-amino acid conjugate hydrolase, aldo/keto reductase, and AILP1. In contrast, Fe(II) oxygenase, senescence-related gene1, and ethylene response factor1, all involved in ethylene response, were induced in concert with the GID1 gene involved in gibberellin signaling. In young leaves, some key upregulated genes were involved with auxins (NGA1), ethylene (flavonol synthase, ethylene-response-element-binding protein), and gibberellin (GASA proteins).

#### Transcription Factors

**FIGURE 5:**
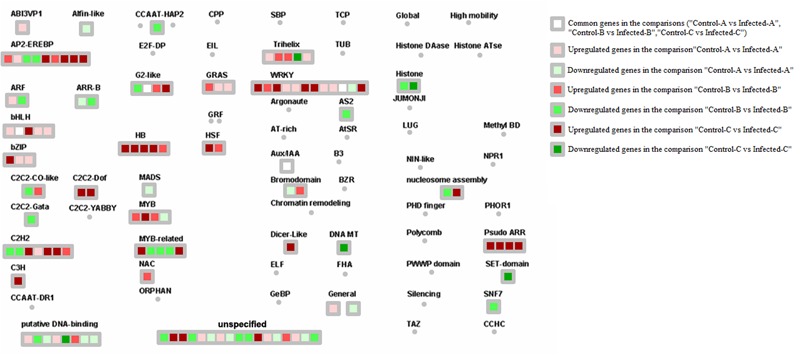
HLB-regulated genes encoding transcription factors. Up- and downregulated genes in the three pairwise comparisons were shown. Common HLB-regulated genes between at least 2 of 3 pairwise comparisons were also shown.

Huanglongbing induced key genes encoding AP2-EREBPs such as SHN1, CRF1, and two AP2 domain-containing transcription factors in young leaves after artificial infection (Fu et al., 2016), another AP2 domain protein and a WRI1 in mature leaves and an ERE-BP in young leaves of symptomatic field trees (**Figure 5**). Other transcription factor categories induced by HLB were HB (Homeobox), MYBs, C2H2, pseudo ARR, and GRAS. HB TFs were induced in young leaves 32 w.a.i., including HB40, HAT9, HB17, and KNAT7. Two MYBs were enhanced in mature leaves (MYB82 and MYB116). Genes encoding C2H2 transcription factors upregulated in young leaves 31 w.a.i. included STZ, zinc finger protein 7 and ZAT10 (Fu et al., 2016). Genes encoding pseudo ARR were also induced: PRR3, PRR5, and PRR7.

WRKY transcription factors are involved in biotic stress responses. Several members of this family were induced by the three RNA-Seq datasets. WRKY60, WRKY70, WRKY40, and WRKY33 were upregulated in young leaves 32 w.a.i. WRKY31 was induced in mature CaLas-infected leaves, while WRKY2, WRKY47, WRKY42, and WRKY7 were enhanced in young leaves of field trees. Only WRKY48 was commonly modulated by CaLas in two of the three datasets.

#### Biotic Stress Responses

**FIGURE 6:**
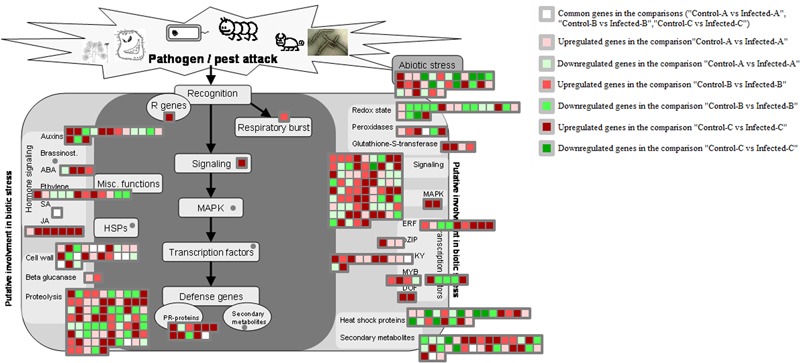
HLB-regulated genes involved in biotic stress responses. HLB-regulated genes in the three pairwise comparisons and commonly modulated between two of the three pairwise comparisons were shown.

Genes encoding glutathione S-transferases such as GST7 and GST25 were generally upregulated by HLB disease in young leaves after artificial infection and in mature CaLas-infected leaves (**Figure 6**). Heme-binding was induced in young leaves, peroxidases were upregulated in mature leaves and two peroxidases were enhanced in artificially infected immature leaves. Genes encoding cell wall modification and restructuring such as GAE6, PRP4, XTR6, RD22, and pectate lyase were upregulated in young leaves after artificial infection. UGE5, CSLG2, RGP2, glycoside hydrolase family 28 proteins, and expansin4 were induced in young leaves.

Seven pathogenesis-related proteins were upregulated in immature leaves (Fu et al., 2016). A transmembrane signaling receptor involved in the SAR response, EDS1, was upregulated in immature leaves after artificial infection (Fu et al., 2016), but not after natural infection (Martinelli et al., 2013).

### Genes Commonly Involved in HLB Response Between Datasets

Genes with the same altered pattern of expression in more than one experiment were identified. More genes involved in primary than secondary metabolism were observed (Supplementary Tables S1, S4).

The category of genes that was most commonly modulated in published transcriptomic datasets was cell wall modification and restructuring (**Figure 3**). These genes were: CESA8, pectinesterase, expansin8, expansin beta 3.1, and pectate lyase. Some other genes linked with more than one transcriptomic study were related to lipids such as brassinosteroid sulfotransferase, myzus persicae-induced lipase 1, and mitochondrial acyl carrier protein 3.

Among genes affecting hormonal crosstalk, an S-adenosylmethionine-dependent methyltransferase was upregulated in multiple transcriptomic datasets. Other key genes involved in biotic stress responses were affected in both young and mature leaves: WRKY48, peroxidase, and F-box family protein. In starch and sucrose metabolism, an invertase was modulated by HLB in multiple studies. Among transcription factors, genes encoding phytochrome interacting 3-like 1, MYB transcription factor, and IAA14 were clearly and specifically linked to HLB response.

### Molecular Mechanisms of HLB Tolerance

The comparison between susceptible and tolerant species highlighted that many more genes involved in photosynthesis and the Calvin cycle were repressed by HLB in susceptible than in tolerant plants (Supplementary Figures S1, S2). However, there were very few genes in common between the HLB response and tolerance datasets. Tolerant genotypes showed downregulation of genes encoding chlorophyll binding, oxygen-evolving complex-related, thylakoid luminal 20 kDa protein, and two ferredoxin-related proteins. Photosystem II reaction PSB28 protein was commonly repressed. In the tolerant genotype, there was a repression of genes involved in tetrapyrrole (NADH-ubiquinone oxidoreductase 20 kDa subunit, alternative, NADH dehydrogenase, ubiquinonl-cytochrome C reductase complex 14 kDa, cytochrome c oxidase, and ATP synthase) and the TCA cycle (LTA2, succinyl-CoA ligase, and malate dehydrogenase). In comparison to HLB response, there were more repressed genes involved in call wall modification such as 4 pectinesterases, PME1, and PME3. There was also decreased transcript abundance of genes involved in cellulose synthesis, cellulases and beta-1,4’glucanases, poligalacturonases, cell wall precursor synthesis, fatty acid synthesis and fatty acid elongation. Genes involved in starch degradation were upregulated, including alpha-amylase2, beta-amylase8, glycoside hydrolase, and starch excess4. Sucrose biosynthesis was enhanced (sucrose-phosphate-synthase, sucrose-phosphate1, and transferase). Secondary metabolism genes involved in terpenes and phenylpropanoids were induced: homogentisate phytyltransferase1, amino_oxidase, carotenoid isomerase, cycloartenol synthase, O-methyltransferase2, and isoflavone-7-O-methyltransferase9. More upregulated genes involved in flavonoid synthesis than in phenylpropanoid synthesis were observed: O-methyltransferase, oxidoreductase, isoflavone reductase, and pinoresinol reductase. More genes involved in amino acid metabolism than biosynthesis were repressed.

#### Hormone Overview

Repression of brassinosteroids and salicylic and jasmonic acid-mediated responses were more linked with tolerance than HLB response. The following genes were downregulated: steroid 5-alpha-reductase, sterol methyl tranferase2, C-8 sterol isomerase, C-5 sterol desaturase, BR1-EMS-suppressor, lipoxygenase, electron carrier, and allene oxide synthase (**Figure 4**). Several genes involved in ethylene-related pathways were upregulated: oxidoreductase, 2-oxoglutarate-dependent dioxygenase, ACS6 and ACS12, HLS1, and ERF104. Several ethylene-related genes were commonly modulated by HLB in the two types of datasets (oxidoreductase, gibberellin-2-beta-dioxygenase, ethylene-regulated nuclear protein, and universal stress protein). A similar number of genes involved in auxin-related pathways were up- or downregulated. While some GA-related genes were upregulated in response, others involved in the same hormone pathways were repressed: GASA4, gibberellin-responsive protein, and GASA protein.

#### Transcription Factors

More MYBs were upregulated in the tolerant genotype: MYB59, MYB55, MYB15, MYB30, MYB73, and MYB52 (**Figure 6**). Other transcription factor categories were induced, including MADS (AGL7, AGL22, and AGL42), B3 DNA binding protein (VRN1), histone ATse (ADA2B, HAF01, and HAC12), C2C2-CO-like (COL9 and zinc finger B-box type), and homeobox (HB-1, HB-7, and HAT9). There were many genes involved in chromatin structure remodeling that were commonly modulated by both response and tolerance: histone4, HTA7, histone H3.2, HMGA, and HTA5.

#### Biotic Stress Responses

In addition to the differentially regulated genes previously mentioned, tolerance was linked with downregulation of genes involved in cellulose and cell wall precursor synthesis such as UDP-glucuronate decarboxylase, UDO-6-glucose-6-dehydrogenase, GDP-mannose 4,6,-dehydratase, rhamnose biosynthesis 1, and cellulose synthase like C4 and D3. Several beta-glucanases were also repressed (Supplementary Figure S3). Twenty-one pathogenesis proteins were induced in tolerant genotypes encoding TIR-NBS-LRR proteins. Detoxifying pathways were upregulated as shown by the induction of several glutathione S-transferases (GSTU19, GST8, GSTU19, GST-TAU20, and GST14). Several genes were commonly modulated in HLB response datasets and the tolerance one: phloem protein 2 A5 (R genes), GSTU7 (detoxifying pathways), TGA1 (bZIP), AIL5, and TINY2 (AP2-EBEPB transcription factors).

#### Large Enzyme Families

Among the large enzyme families, upregulation of glutathione S-transferases was linked with tolerance, as were oxidases (copper amine oxidase, NADP-dependent oxidoreductase, flavin-containing monooxygenase, and CTF2A). Genes involved in cytochrome P450-related reactions were more upregulated than downregulated in tolerant genotypes than in susceptible ones. There was general repression of glucosidases, beta-1,3-glucan hydrolases, GDSL-lipases, and nitrilases. Several key genes belonging to large enzyme families were commonly regulated between tolerance and response: FAD-binding domain containing protein, glucose-methanol-choline, MES17, and two peroxidases.

### Protein–Protein Network Analysis

**FIGURE 7:**
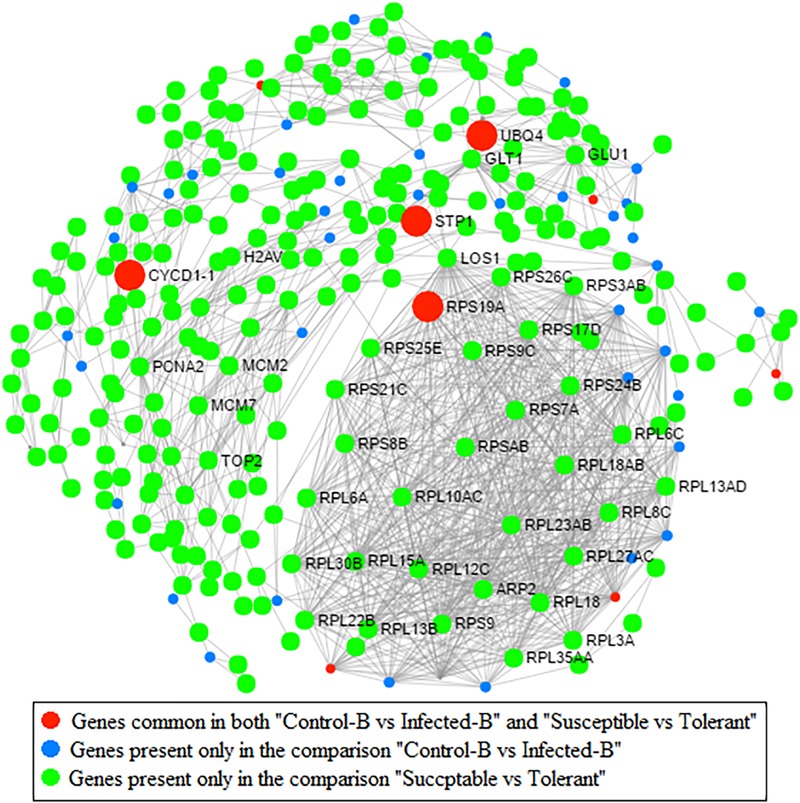
Protein–protein network analysis based on Arabidopsis knowledge base. Genes commonly modulated between datasets from Martinelli et al. (2013) (HLB response) and Wang et al. (2016) (HLB tolerance) are indicated. Genes present only in the comparison between healthy and infected in young leaves are also indicated (dataset B; Martinelli et al., 2013). Genes present in the comparison between susceptible vs. tolerant genotype are shown (Wang et al., 2016).

Protein–protein interaction (PPI) network analysis based on an Arabidopsis knowledgebase compaired two pairwise comparisons performed on the same type of leaf tissue (young leaves): one related to HLB response (dataset B) and one linked with HLB tolerance (dataset D) (**Figure 7**). Four highly interactive proteins encoded by genes commonly regulated between the two datasets were identified: UBQ4, CYCD1-1, RPS19A, and STP1. A second PPI network analysis was performed to identify proteins commonly modulated by HLB in the three HLB-response datasets A, B, and C and HLB tolerance dataset D (Supplementary Figure S4). Only a CSD2 protein was commonly present in the four pairwise comparisons. The comparison between the three leaf RNA-Seq datasets involved in HLB response showed that three heat shock proteins (HSP70-5, HSFB1, and HSP25.3) were encoded by genes that were significantly regulated in all three datasets.

### Splice Analysis

**FIGURE 8:**
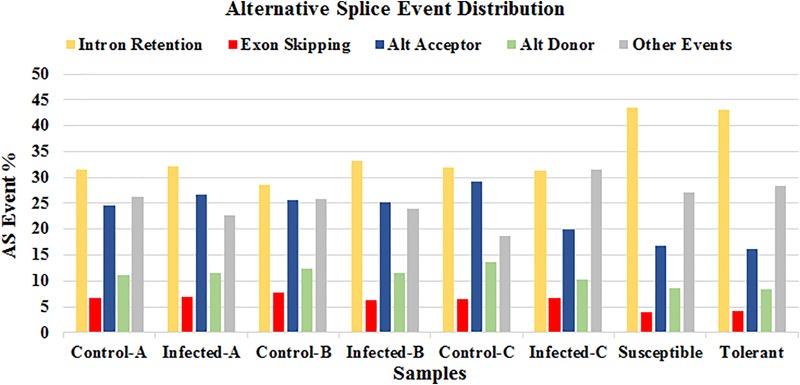
Distribution of the predicted splice events by ASTALAVISTA online tool for each samples. The splice categories are intron retention (IR), alternative splice donor (AD), alternative splice acceptor (AA), exon skipping (ES) and the combination of the above mentioned splice mechanisms (Other Events).

The online tool ASTALAVISTA^3^ predicted the splice events intron retention (IR), alternative splice donor (AD), alternative splice acceptor (AA), exon skipping (ES), and the combination of the above mentioned splice mechanisms from the eight samples. We observed that the splice event IR is the most abundant type (28.5–43.4%), followed by AA (16.1–29.1%), AD (8.3–13.6%) (**Figure 8** and Supplementary Table S5). A considerable amount of the combination of splice events (classified as ”Other Events,” 18.7–31.6%) were also observed from all the samples.

The complete AS events (AS landscapes) identified in this study can be downloaded from the link https://drive.google.com/drive/folders/1oorwtZmEcwSAs1n6x4g_fEGS-HOrxIbK?usp=sharing.

The data contain the information of exon-intron structure of the AS events, chromosomal coordinates, the IDs of the transcripts, involved in the given AS event. For the exon-intron structure of the AS event, ES is indicated by 1–2^∧^,0, alternative donor (AD) by 1^∧^,2^∧^, alternative acceptor (AA) by 1-,2- and IR by 1^∧^2-,0^8^.

The differential regulations of alternative spliced forms of the commonly modulated genes between the four datasets were observed in response to HLB. The quantitative visualization of splice junction of the genes showing significant psi score difference was done using “sashimi_plot” program in MISO (Mixture of Isoforms) tool along with uninfected sample as sashimi plot (Supplementary Figures S5–S7 and Supplementary Table S5). The sashimi plot shows the number of reads corresponding to specific exon–exon junctions was labeled for each junction. We observed higher bayes factor for the splice event ‘orange1.1g023621m.g.v1.1’ in all datasets except “Control-C vs. Infected-C”, which showed that the isoform is more likely to be differentially expressed (Supplementary Table S5). Exon-skipping events found for the splice event ‘orange1.1g010747m.g.v1.1’ in samples ‘Control-A’ and ‘Control-C’. The splicing event ‘orange1.1g021628m.g.v1.1’ was not detected in the control sample ‘Control-C.’

The MISO differential expression result files for each comparison were given below.

Control-A vs. Infected-A: https://drive.google.com/open?id=1caFTINtMPt2YAcS8IP7hNTRPpxsIBbk4
Control-B vs. Infected-B: https://drive.google.com/open?id=1g6PLt8sG3_RRs0LlZ8BwDNibczYvCSzs
Control-C vs. Infected-C: https://drive.google.com/open?id=1rW1o7JIifvE2Z00KS8z7cljb0k_tV6Uq
Susceptible vs. Tolerant: https://drive.google.com/open?id=17EhM6q96uDBrcO4wpij5xA96cKardt-x

Multivariate analysis of transcript splicing (MATS) provides a statistical framework that determines the junction counts supporting the inclusion or the exclusion of specific splice events in Treated sample against Control. We ran MATS for all four comparisons and extracted the AS events only for the common genes. Only one gene ‘orange1.1g023621m.g.v1.1’ reported AS event alternative 5′ splice site (A5SS) in the rMATS results of all four comparisons (Supplementary Table S5) and reported a skipped exon (SE) AS event only in the comparison “Susceptible vs. Tolerant.”

The rMATS result files for each comparison were given below.

Control-A vs. Infected-A: https://drive.google.com/open?id=1j-1JaOolzZAiqCbBYoj8Fq8UYGF23V4q
Control-B vs. Infected-B: https://drive.google.com/open?id=1Yy-zF_g3gq09Hd15cC6vYUtB2A9ik523
Control-C vs. Infected-C: https://drive.google.com/open?id=1QEQvoZZ4qGRCLpBKhvXh-yVnUoBHJXxd
Susceptible vs. Tolerant: https://drive.google.com/open?id=1Rvqbx7KF5-LDVN5APZw9qvU-bk4MXkuy

## Discussion

The aim of this work was to identify genes and pathways commonly modulated by HLB disease in different published RNA-Seq datasets examining leaf tissues and tolerance mechanisms. The high variability in transcriptomic data requires more bioinformatic analysis. Most transcriptomic studies on HLB response were performed in only one season and using different agronomic, developmental and physiological conditions, weakening data reliability. Our work compared all available RNA-Seq datasets related to HLB responses in Citrus leaf tissues. First, we compared three transcriptomic datasets performed on leaves infected by CaLas and then we sought common findings between these three studies and one examining HLB tolerance.

Although repression of photosynthesis and upregulation of starch and sucrose-related genes were observed in all three leaf datasets, few genes were commonly regulated. These data agreed with published findings that genes involved in photosynthetic reactions are generally downregulated by HLB disease (Albrecht and Bowman, 2008; Kim et al., 2009; Martinelli et al., 2013). The comparison between the study on artificial infections (Fu et al., 2016) and that performed under field conditions (Martinelli et al., 2013) showed that different genes may have contrasting expression trends in the same tissue, young leaves. This implies that experimental conditions may drastically affect data, leading to contrasting conclusions.

The present study showed how different variables (developmental, agronomic and physiological conditions, and infection method) affect expression of key genes in primary metabolism. Some common features between the three transcriptomic datasets involved cell wall modifications. Six genes involved in cell wall-related pathways were commonly modulated by HLB in all three leaf datasets. We speculate that these genes may affect plant signaling responses to CaLas infection because of the role played by cell wall restructuring in sensing pathogen infections (Corwin and Kliebenstein, 2017).

Sugar and starch metabolism has been linked to a possible pathogenetic mechanism of CaLas (Martinelli and Dandekar, 2017). The induction of genes involved in sucrose degradation (invertase), starch biosynthesis (starch branching enzyme and starch synthase), and starch degradation (amyl-amylase, beta-amylase, and phosphoglucan water dikinase) were clearly induced by HLB in leaves (Albrecht and Bowman, 2008; Martinelli et al., 2013). Starch accumulation is a clear symptom of HLB progression in leaves (Bove, 2006). Unfortunately, our work found no HLB-induced gene involved in starch metabolism that was commonly modulated in different datasets. Only a beta-fructosidase involved in sucrose degradation was commonly modulated by HLB in the three datasets, perhaps due to the many differences in physiological, developmental, environmental, and agronomic conditions between the two studies (Martinelli et al., 2013; Fu et al., 2016). These findings confirmed the difficulty in finding common, specific host biomarkers to complement traditional diagnostic approaches relying on pathogen detection. Further works on RNA-Seq studies will demonstrate whether key markers can identify natural CaLas infections under field conditions.

Among all the hormone categories, only S-adenosylmethionine-dependent methyltransferase was commonly modulated by HLB in at least two of the three leaf datasets. Although more genes involved in hormonal crosstalk were expected be commonly regulated among studies, this evidence highlighted that SAR responses were activated in leaf tissues: an expected result, since CaLas is a biotrophic pathogen. The upregulation of several genes involved in jasmonic-mediated responses (lox1, lox2, and lox3) confirmed that typical defense responses against necrotrophic pathogens are induced by CaLas infection. A possible pathogenetic mechanism of CaLas is its modulation of hormonal-mediated defense responses for its own benefit (Martinelli et al., 2012, 2013; Martinelli and Dandekar, 2017). Although MYC2, a gene involved in jasmonic acid inhibition of salicylic acid responses, was not altered in any dataset, this present work confirmed a possible role for jasmonic acid in counteracting SAR responses in CaLas-infected leaves (Kazan and Manners, 2013). Abscissic acid and auxin genes can negatively affect SAR responses (Pieterse et al., 2009). Although the upregulation of key genes involved in auxin biosynthesis, metabolism and response in artificially infected young leaves may inhibit salicylic acid responses, these genes were not affected in leaves under field conditions. Other auxin genes such as GH3.1, GH3.9, and GH3.17 were induced (Martinelli et al., 2013). That no commonly regulated genes were found among the three transcriptomic datasets is another illustration of the high variability of transcriptomic data taken under different experimental conditions. Data obtained by Fu et al. (2016) highlighted the induction of two key genes involved in abscissic acid responses (GRAM domain containing protein and benzodiazepine receptor-related). Another gene, HVA22, was induced in field-grown young leaves. Taken together, these findings suggest that abscissic acid-related genes may aid pathogen colonization of the Citrus plant. The positive effect of gibberellins on SAR response is well known (Pieterse et al., 2009). Two genes involved in gibberellin pathways were upregulated in field-grown mature leaves and one was induced in young leaves in the same study (Martinelli et al., 2013). Another hydrolase potentially involved in auxin pathways was induced in immature leaves (Fu et al., 2016). Taken together, these findings suggest that upregulation of gibberellin-related genes may compensate for negative effects of ABA and auxin on the SAR response. No gene involved in brassinosteroid was modulated by HLB in infected leaves. This contradicts findings that highlight the involvement of brassinosteroid-related genes on biotic stress responses.

Among transcription factors, HB, AP2EREBP, and Pseudo ARR were mostly induced in the dataset of Fu et al. (2016) while GRAS and bHLH were upregulated in leaves analyzed by Martinelli et al. (2013). This evidence highlighted again how different experimental conditions affect expression of different key genes involved in CaLas responses. WRKYs are a family of transcription factors mostly involved in environmental plant stress responses (Jiang et al., 2016; Balan et al., 2017, 2018). Although only WRKY48 was commonly regulated between the three transcriptomic leaf datasets, several WRKYs were highly upregulated by HLB disease: five genes were induced in artificially infected young leaves, four in immature field-grown leaves and one in mature field-grown leaves. Because hundreds of different WRKYs are documented in crops (Rushton et al., 2010; Fan et al., 2015), only one WRKY may be involved in any specific biotic or abiotic stress. Specific WRKYs are induced by almost all environmental stresses and their expression is often tissue-specific (Jiang et al., 2016). However, analysis of this group of transcription factors clearly linked to environmental stress may help early diagnosis of CaLas infections, when HLB disease is at an early stage. Although this must be confirmed by further experiments focusing on early disease stages, plant diagnostic approaches relying on host responses have been proposed (Ibáñez et al., 2014; Martinelli et al., 2016). This approach may complement traditional diagnostic methods based on PCR that target the pathogen, but will not replace them. This approach may be particularly helpful for plant diseases characterized by long incubation times.

Some genes involved in glutathione S-transferases were HLB-modulated in all three analyzed leaf datasets. More genes encoding pathogenesis-related proteins were upregulated in the datasets of Fu et al. (2016) than in the one obtained by Martinelli et al. (2013). Artificial infection may induce a stronger response in infected Citrus than natural infection. This is also confirmed by the upregulation of EDS1 after artificial, but not natural, infection. This gene is the receptor for salicylic acid-mediated responses (Parker et al., 1996). We speculate that the infection method (artificial or natural) deeply affects host responses to pathogen attack, driving diverse hormone-mediated defense responses.

Protein–protein interaction network analysis was conducted to identify which HLB-modulated genes play a key role at the PPI level in both HLB response and tolerance. The identification of three heat shock proteins commonly modulated between the three leaf HLB-response datasets confirmed their key role in disease progression and symptomatology (Martinelli et al., 2012, 2013; Martinelli and Dandekar, 2017).

This bioinformatic analysis highlights how different transcriptomic studies dealing with the same subject tend to show few commonly regulated genes. This may be due to the high environmental variability of field studies, leading to large differences in physiological and environmental conditions. However, identification of common features between studies helps clarify the role of CaLas in this devastating Citrus disease. More works will be useful once more RNA-Seq datasets are available. An analysis comparing microarray and RNA-Seq data is highly desirable.

This manuscript highlights how different transcriptomic studies dealing with the same subject tend to show few commonly regulated genes. This may be due to the high environmental variability of field studies, leading to large differences in physiological and environmental conditions. Metabolomic approaches integrating transcriptome data on plant stress responses will be highly desirable as those previous employed (Rizzini et al., 2010; Tosetti et al., 2012; Saia et al., 2015). However, identification of common features between studies helps clarify the role of CaLas in this devastating Citrus disease. More works will be useful once more RNA-Seq datasets are available. A new bioinformatic analysis comparing microarray and RNA-Seq data is highly desirable such as previously performed in Citrus (Martinelli et al., 2015).

## Author Contributions

BB performed the data analysis using the described the functional genomic, bioinformatics tools. FM conceived, designed the research work and mainly wrote the article. BB, AI, AD, TC, and FM significantly contributed on writing the manuscript.

## Conflict of Interest Statement

The authors declare that the research was conducted in the absence of any commercial or financial relationships that could be construed as a potential conflict of interest.

## Footnotes

1 https://phytozome.jgi.doe.gov

2 https://phytozome.jgi.doe.gov/pz/portal.html

3 http://genome.crg.es/astalavista/

4 http://genes.mit.edu/burgelab/miso/docs/

5 http://rnaseq-mats.sourceforge.net/

6 http://mapman.gabipd.org/

7 https://david.ncifcrf.gov/

8 http://genome.crg.es/astalavista/FAQ.html
