# Supplementary material for: Identifying Host Molecular Features Strongly Linked With Responses to Huanglongbing Disease in Citrus Leaves
Source: Front Plant Sci. 2018 Feb 28;9:277. doi: 10.3389/fpls.2018.00277 (PMC5836289; doi:10.3389/fpls.2018.00277)
Supplement: Supplementary file 6 [file Image_1.pdf]

Supplementary Material

Identifying host molecular features strongly linked with responses to Huanglongbing disease in Citrus leaves

Bipin Balan[1], Ana M. Ibáñez[2], Abhaya M. Dandekar[2], Tiziano Caruso[1], Federico Martinelli[1]*

[1]Dipartimento di Scienze Agrarie Alimentari e Forestali, Università degli Studi di Palermo, Palermo, Italy.

[2]Plant Sciences Department, University of California Davis, Davis, California, United States of America

*** Correspondence:**

Federico Martinelli

federico.martinelli@unipa.it

Supplementary Figures

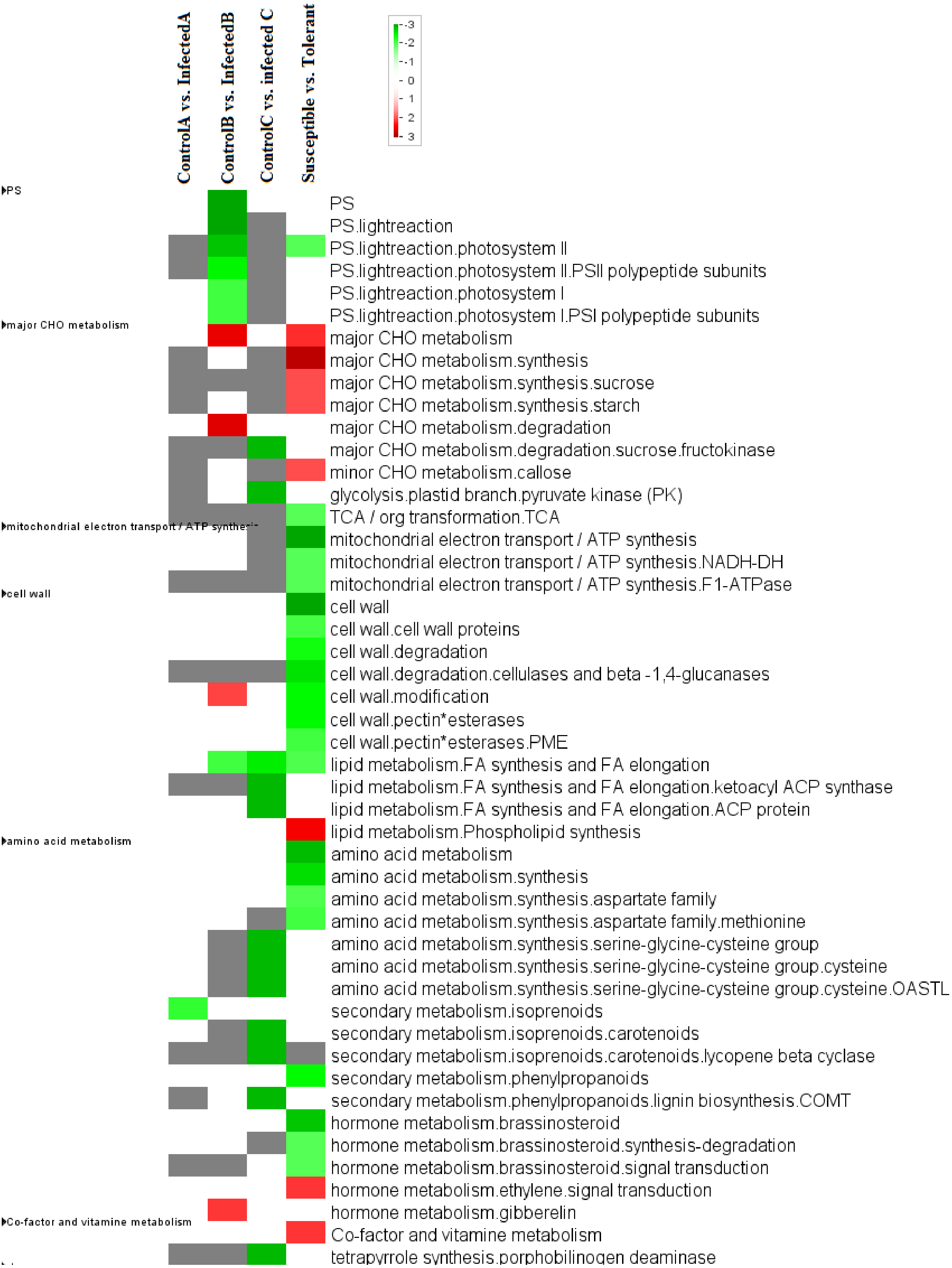

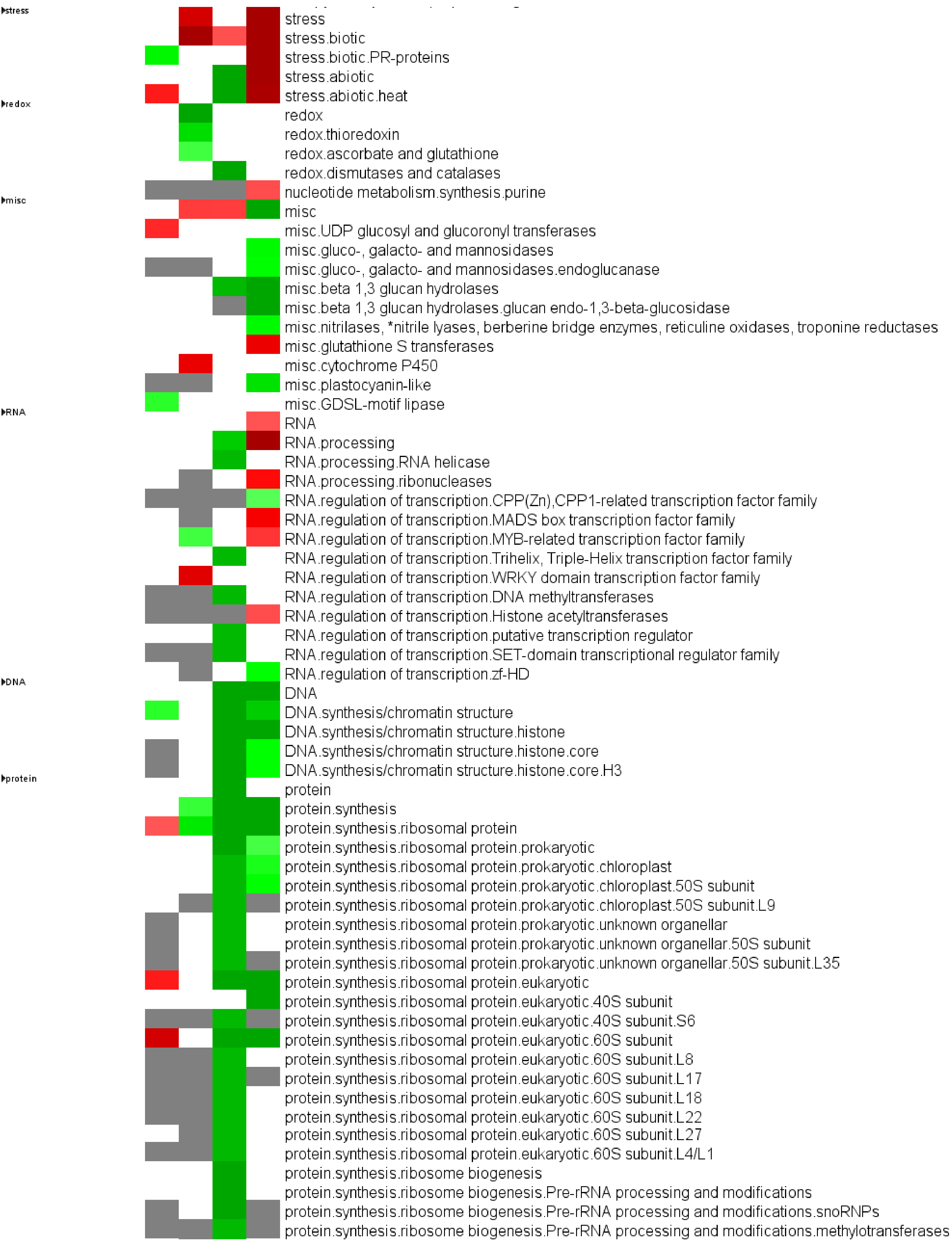
stress
redox
misc
RNA
DNA
protein
stress
stress.biotic
stress.biotic.PR-proteins
stress.abiotic
stress.abiotic.heat
redox
redox.thioredoxin
redox.ascorbate and glutathione
redox.dismutases and catalases
nucleotide metabolism.synthesis.purine
misc
misc.UDP glucosyl and glucoronyl transferases
misc.gluco-, galacto- and mannosidases
misc.gluco-, galacto- and mannosidases.endoglucanase
misc.beta 1,3 glucan hydrolases
misc.beta 1,3 glucan hydrolases.glucan endo-1,3-beta-glucosidase
misc.nitrilases, *nitrile lyases, berberine bridge enzymes, reticuline oxidases, troponine reductases
misc.glutathione S transferases
misc.cytochrome P450
misc.plastocyanin-like
misc.GDSL-motif lipase
RNA
RNA.processing
RNA.processing.RNA helicase
RNA.processing.ribonucleases
RNA.regulation of transcription.CPP(Zn),CPP1-related transcription factor family
RNA.regulation of transcription.MADS box transcription factor family
RNA.regulation of transcription.MYB-related transcription factor family
RNA.regulation of transcription.Trihelix, Triple-Helix transcription factor family
RNA.regulation of transcription.WRKY domain transcription factor family
RNA.regulation of transcription.DNA methyltransferases
RNA.regulation of transcription.Histone acetyltransferases
RNA.regulation of transcription.putative transcription regulator
RNA.regulation of transcription.SET-domain transcriptional regulator family
RNA.regulation of transcription.zf-HD
DNA
DNA.synthesis/chromatin structure
DNA.synthesis/chromatin structure.histone
DNA.synthesis/chromatin structure.histone.core
DNA.synthesis/chromatin structure.histone.core.H3
protein
protein.synthesis
protein.synthesis.ribosomal protein
protein.synthesis.ribosomal protein.prokaryotic
protein.synthesis.ribosomal protein.prokaryotic.chloroplast
protein.synthesis.ribosomal protein.prokaryotic.chloroplast.50S subunit
protein.synthesis.ribosomal protein.prokaryotic.chloroplast.50S subunit.L9
protein.synthesis.ribosomal protein.prokaryotic.unknown organellar
protein.synthesis.ribosomal protein.prokaryotic.unknown organellar.50S subunit
protein.synthesis.ribosomal protein.prokaryotic.unknown organellar.50S subunit.L35
protein.synthesis.ribosomal protein.eukaryotic
protein.synthesis.ribosomal protein.eukaryotic.40S subunit
protein.synthesis.ribosomal protein.eukaryotic.40S subunit.S6
protein.synthesis.ribosomal protein.eukaryotic.60S subunit
protein.synthesis.ribosomal protein.eukaryotic.60S subunit.L8
protein.synthesis.ribosomal protein.eukaryotic.60S subunit.L17
protein.synthesis.ribosomal protein.eukaryotic.60S subunit.L18
protein.synthesis.ribosomal protein.eukaryotic.60S subunit.L22
protein.synthesis.ribosomal protein.eukaryotic.60S subunit.L27
protein.synthesis.ribosomal protein.eukaryotic.60S subunit.L4/L1
protein.synthesis.ribosome biogenesis
protein.synthesis.ribosome biogenesis.Pre-rRNA processing and modifications
protein.synthesis.ribosome biogenesis.Pre-rRNA processing and modifications.snoRNPs
protein.synthesis.ribosome biogenesis.Pre-rRNA processing and modifications.methylotransferases

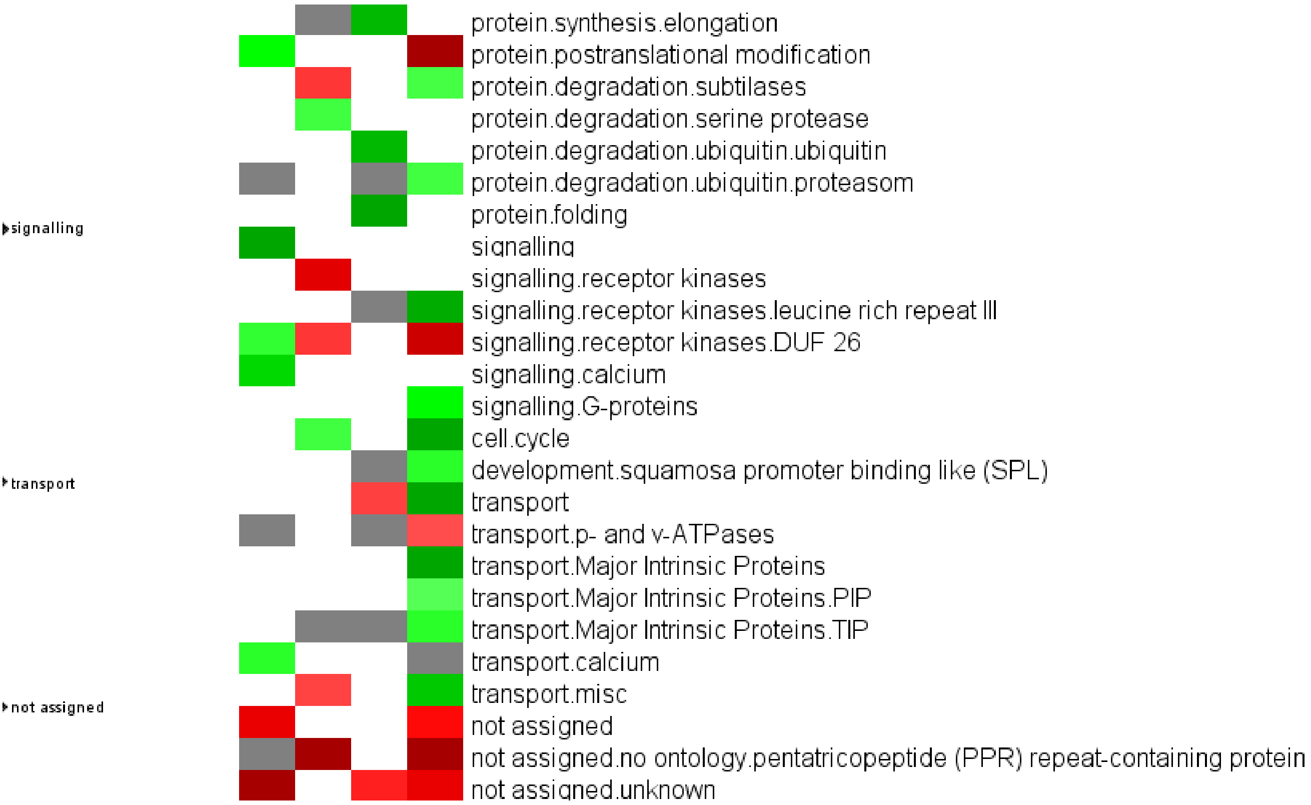

Fig. S1. Gene set enrichment analysis of the four RNA-Seq datasets using Pageman software. Red indicates an over-represented gene set in the differentially regulated gene list compared with total genes present in the mapping file (upregulation) while green means an under-represented gene set category (downregulation).

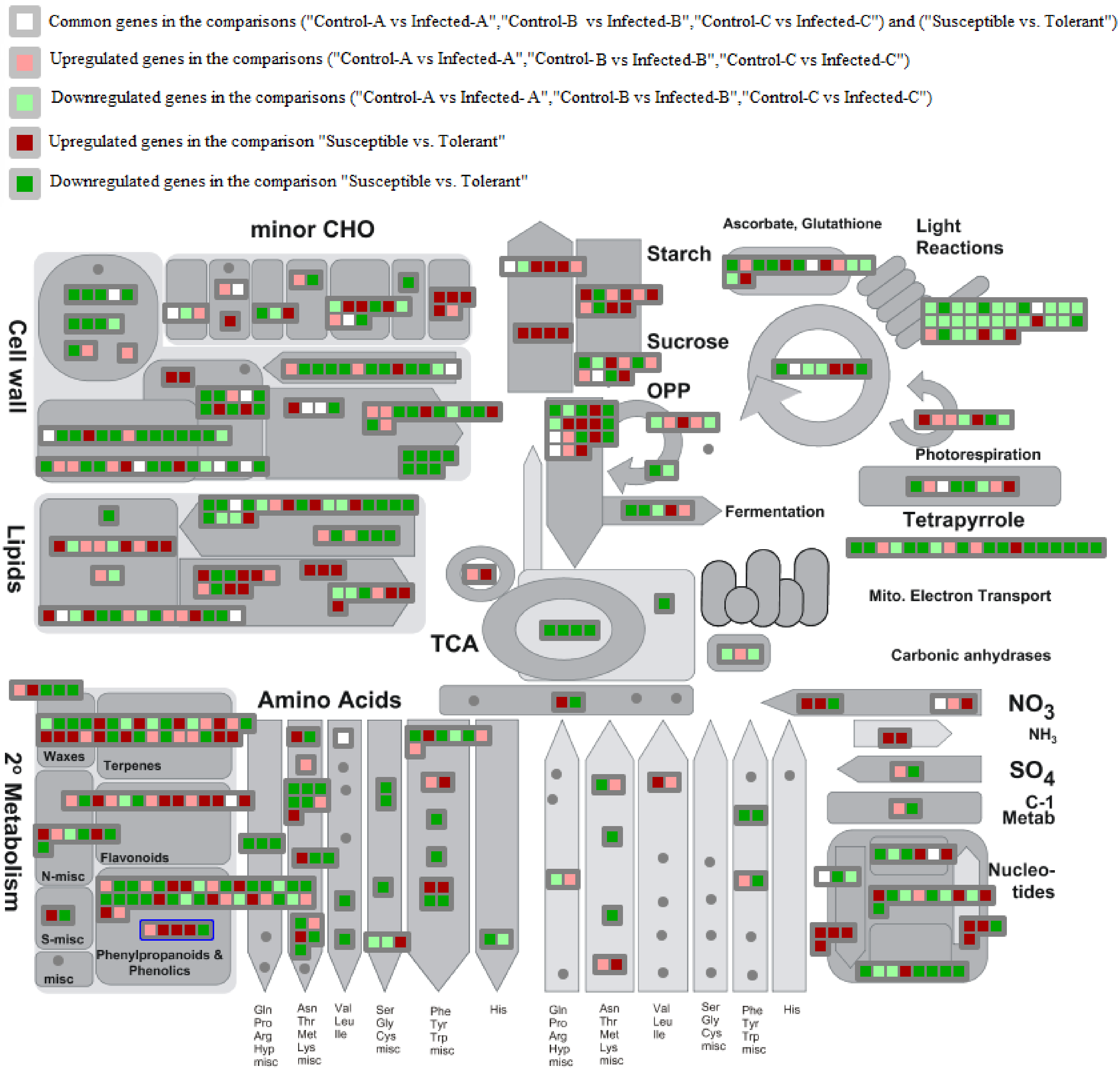

Fig. S2. Mapman overview showing differentially regulated genes between susceptible and tolerant genotypes. Genes commonly modulated between this dataset and the three related to HLB response are shown.

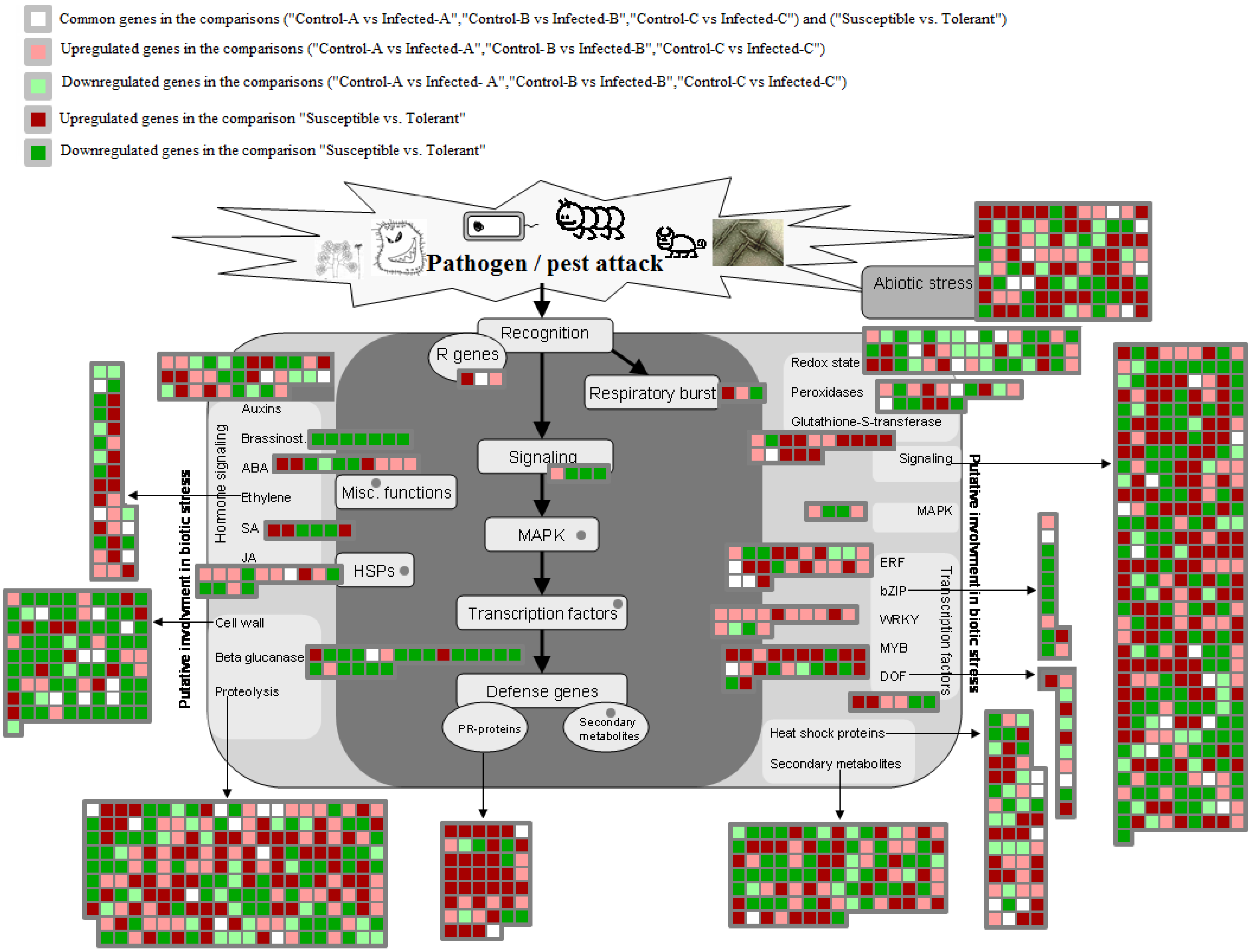

Fig. S3. Biotic stress response genes showing differentially regulated genes between susceptible and tolerant genotypes. Genes commonly modulated between this dataset and the three related to HLB response are shown.

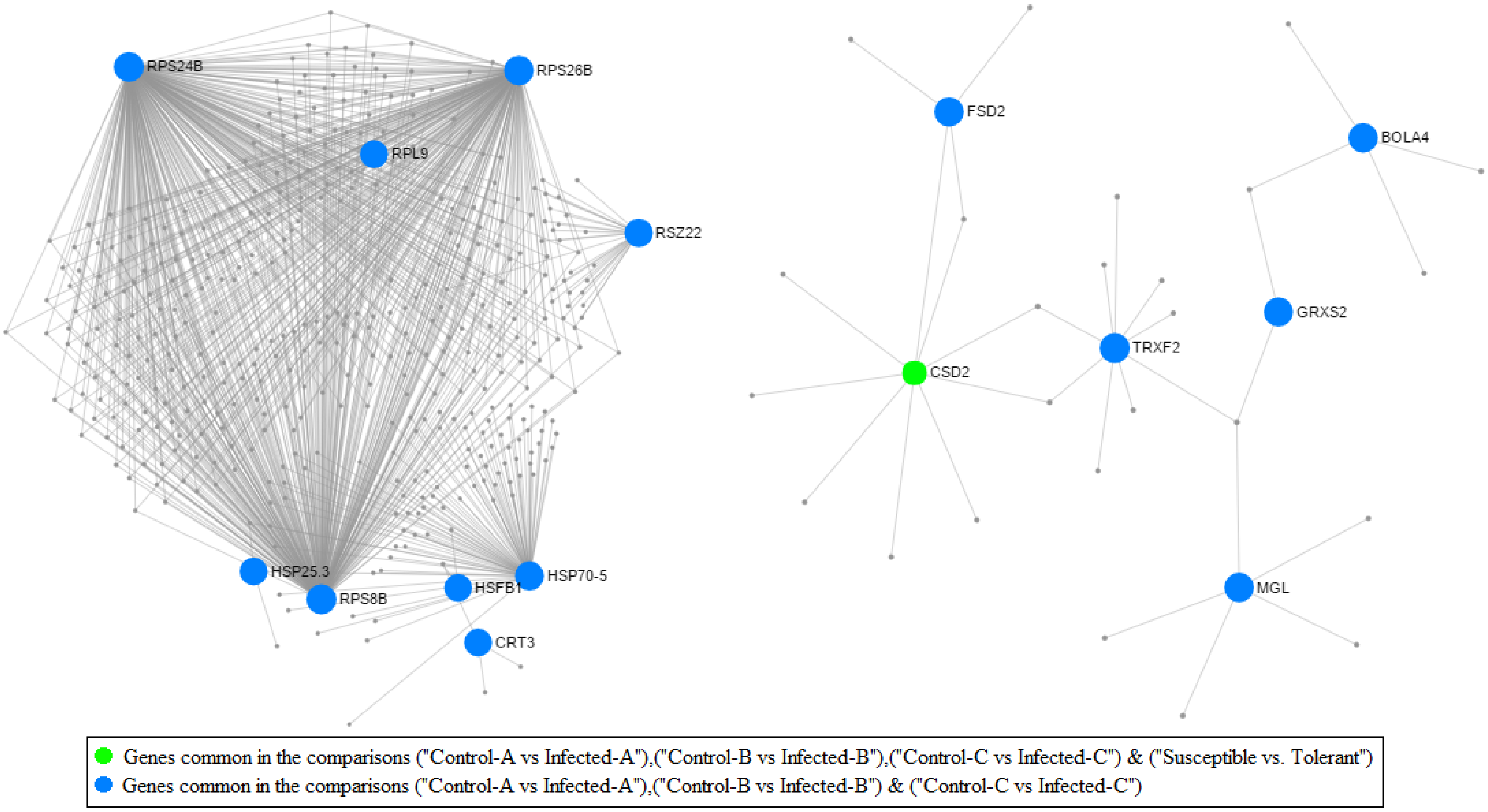

Fig. S4. Protein-protein network analysis based on Arabidopsis knowledge base. Genes commonly modulated between the three RNA-Seq datasets related to HLB response and all four datasets.

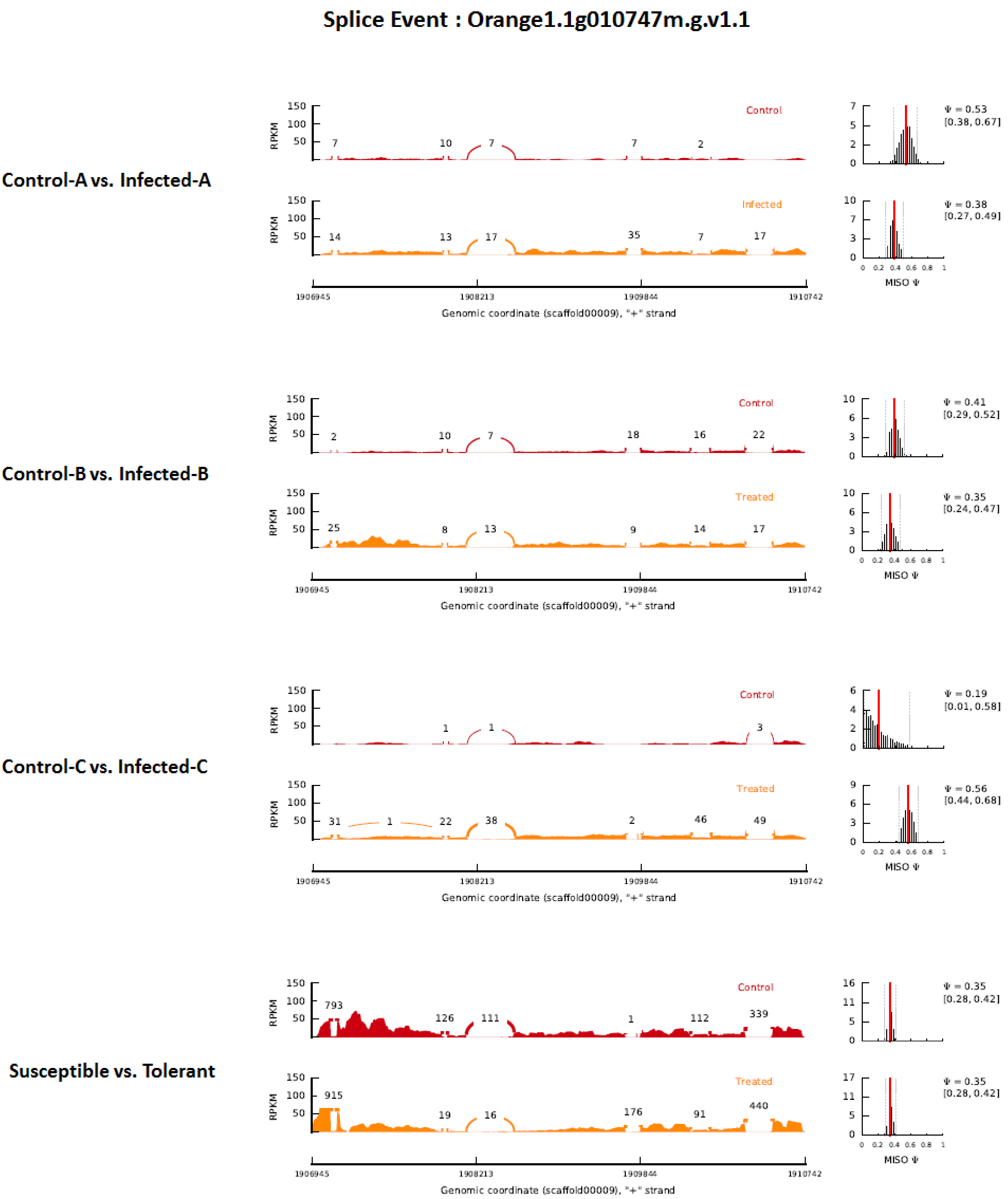

Fig. S5. Splice event 'Orange1.1g010747m.g.v1.1'. The Sashimi plots of the splice event Orange1.1g010747m.g.v1.1 in all four datasets were plotted.

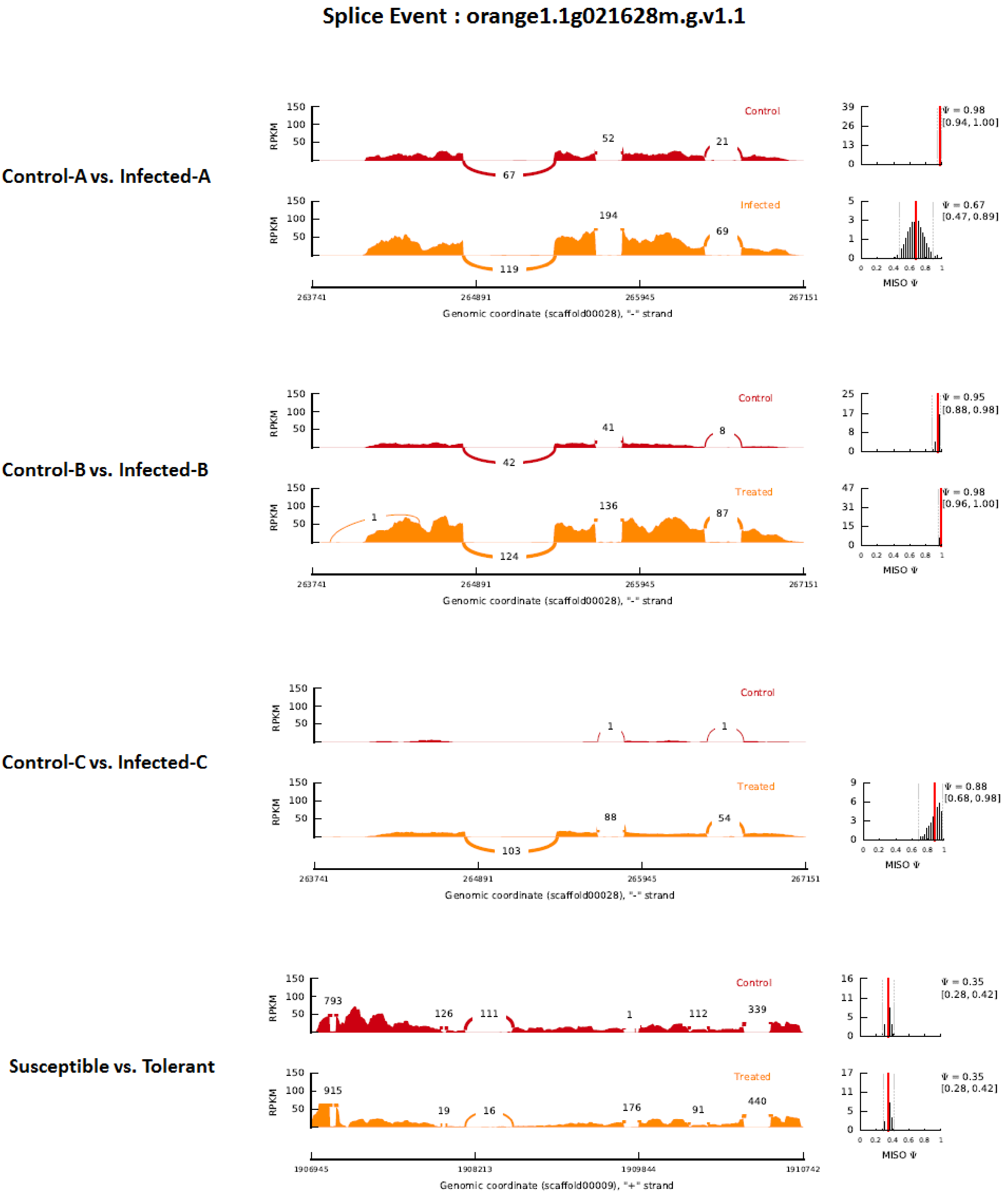

Fig. S6. Splice event 'Orange1.1g021628m.g.v1.1'. The Sashimi plots of the splice event Orange1.1g021628m.g.v1.1 in all four datasets were plotted.

Splice Event : orange1.1g023621m.g.v1.1

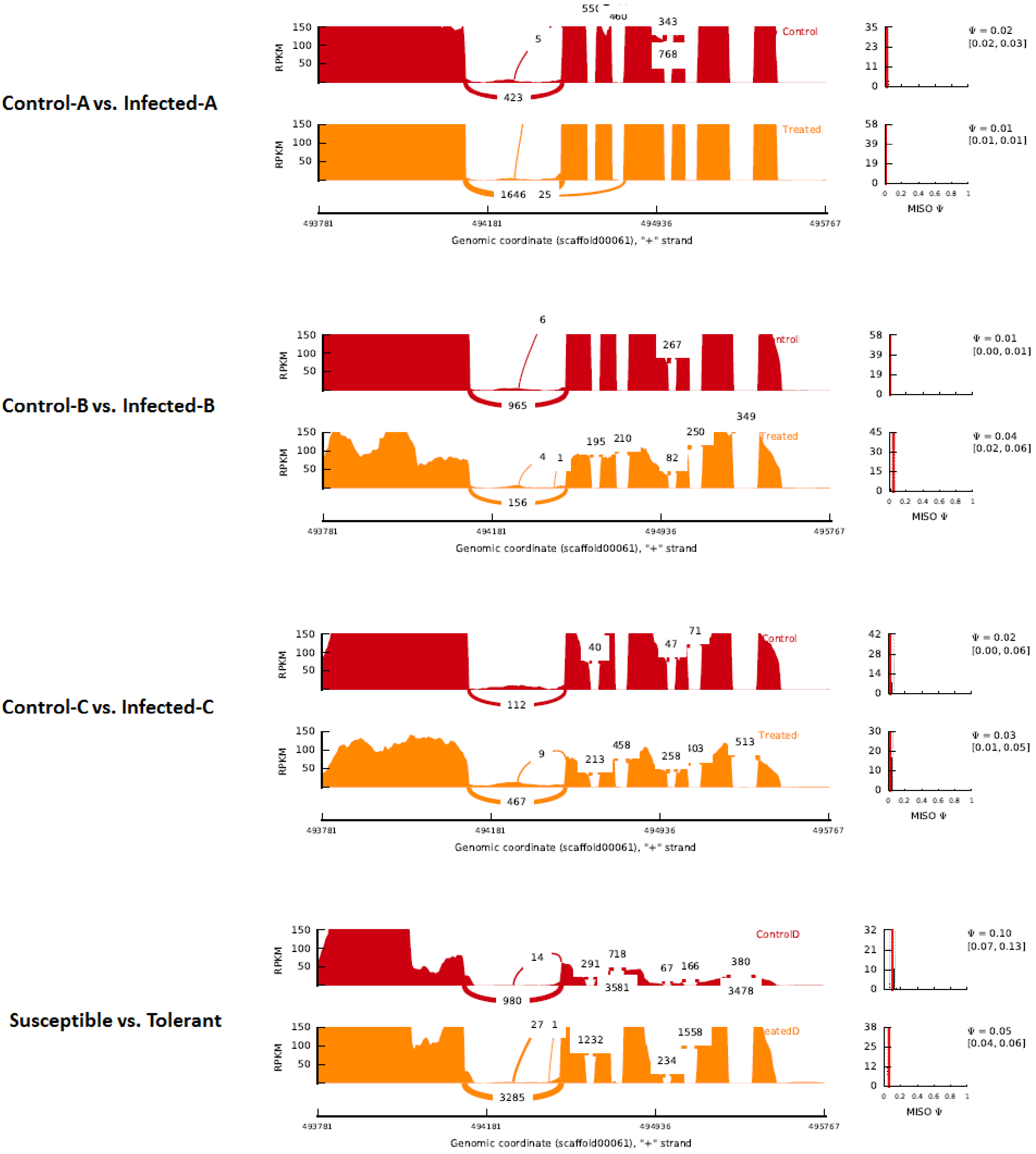

Fig. S7. Splice event 'Orange1.1g023621m.g.v1.1'. The Sashimi plots of the splice event Orange1.1g023621m.g.v1.1 in all four datasets were plotted.
